# Radiomic Analysis of Tumour Heterogeneity Using MRI in Head and Neck Cancer Following Chemoradiotherapy: A Feasibility Study

**DOI:** 10.3389/fonc.2022.784693

**Published:** 2022-02-15

**Authors:** Amrita Guha, Mustafa Anjari, Gary Cook, Vicky Goh, Steve Connor

**Affiliations:** ^1^ Department of Radio-Diagnosis, Tata Memorial Hospital, Mumbai, India; ^2^ Training School Complex, Homi Bhabha National Institute, Mumbai, India; ^3^ School of Biomedical Engineering & Imaging Sciences, Faculty of Life Sciences & Medicine, King’s College London, London, United Kingdom; ^4^ Department of Radiology, Guy’s and St Thomas’ Hospital, London, United Kingdom; ^5^ King’s College London & Guy’s and St Thomas’ Positron Emission Tomography (PET) Centre, London, United Kingdom; ^6^ Department of Neuroradiology, King’s College Hospital, London, United Kingdom

**Keywords:** radiomics, heterogeneity, head and neck cancer, chemoradiotherapy, texture analysis, magnetic resonance imaging

## Abstract

**Objectives:**

To evaluate interval changes in heterogeneity on diffusion-weighted apparent diffusion coefficient (ADC) maps and T1-weighted post-gadolinium (T1w post gad) MRI in head and neck carcinoma (HNSCC), with and without chemo-radiotherapy (CRT) response.

**Methods:**

This prospective observational cohort study included 24 participants (20 men, age 62.9 ± 8.8 years) with stage III and IV HNSCC. The primary tumour (n = 23) and largest lymph node (n = 22) dimensions, histogram parameters and grey-level co-occurrence matrix (GLCM) parameters were measured on ADC maps and T1w post gad sequences, performed pretreatment and 6 and 12 weeks post CRT. The 2-year treatment response at primary and nodal sites was recorded. The Wilcoxon signed-rank test was used to compare interval changes in parameters after stratifying for treatment response and failure (p < 0.001 statistical significance).

**Results:**

23/23 primary tumours and 18/22 nodes responded to CRT at 2 years. Responding HNSCC demonstrated a significant interval change in ADC histogram parameters (kurtosis, coefficient of variation, entropy, energy for primary tumour; kurtosis for nodes) and T1w post gad GLCM (entropy and contrast in the primary tumour and nodes) by 6 weeks post CRT (p < 0.001). Lymph nodes with treatment failure did not demonstrate an interval alteration in heterogeneity parameters.

**Conclusions:**

ADC maps and T1w post gad MRI demonstrate the evolution of heterogeneity parameters in successfully treated HNSCC by 6 weeks post CRT; however, this is not observed in lymph nodes failing treatment.

**Advances in Knowledge:**

Early reduction in heterogeneity is demonstrated on MRI when HNSCC responds to CRT.

## Introduction

Head and neck squamous cell carcinoma (HNSCC) is the seventh most common cancer worldwide (1). Chemo-radiotherapy (CRT) provides the best opportunity for cure in advanced-stage HNSCC (2); however, tumour resistance or insufficient therapy may result in treatment failure in more than 30% of patients (3). Whilst earlier detection of residual viable tumour allows for salvage surgery and improved survival (4), it is currently challenging to evaluate this with clinical examination and cross-sectional imaging, due to the presence of posttreatment tissue distortion (5). Metabolic imaging with 18F-fluorodeoxyglucose positron emission tomography/computed tomography (^18^F-FDG PET/CT) (6) may overcome some of the difficulties in interpretation with conventional CT and MRI in this clinical context, but it is generally delayed for at least 12 weeks, due to earlier false positives from post-CRT inflammation (7).

Assessing changes in MRI signal heterogeneity within tumours (8, 9) may better reflect residual disease. There are limited studies addressing the application of such analysis of imaging features in the prediction of treatment response (10, 11), and there are no data on their early posttreatment evolution in HNSCC. It is well accepted that HNSCCs display a marked “heterogeneity” on histology with variation in proliferation and cellular differentiation within different regions of the tumours (12, 13). We hypothesised that alterations in tumour heterogeneity following CRT would be reflected by diffusion-weighted and post-gadolinium T1w MRI and that measurements of signal heterogeneity may augment standard response assessment based on size criteria alone.

Therefore, our primary objective was to evaluate the interval changes in signal heterogeneity on diffusion-weighted (DWI) apparent diffusion coefficient (ADC) maps and T1-weighted post-gadolinium (T1w post gad) MRI within the primary tumour and largest metastatic lymph node at 6 and 12 weeks following CRT. Our secondary objectives were to evaluate how interval changes vary according to treatment response and to compare the interval changes in parameters to those of conventional size criteria.

## Materials and Methods

### Study Design

Participants were recruited for a prospective single-centre cohort observational study (ISRCTN58327080; Research Ethics Committee approval 13/LO/1876) and provided informed consent for participation.

### Participants

Participants were eligible if there was histologically confirmed stage III or IV primary squamous cell carcinoma of the head and neck (HNSCC) without distant metastatic disease, a 1-cm^2^ area of measurable primary tumour and/or metastatic locoregional node on the basis of standard clinico-radiological staging, and where curative CRT was planned. Exclusion criteria were prior chemo- or radiotherapy; ECOG performance status >2; lack of capacity to provide informed consent; and known contrast agent allergy or renal impairment.

### Treatment

Intensity-modulated radiotherapy consisting of 70 Gy in 35 fractions was delivered, 2 Gy per fraction once daily, 5 days a week. Concomitant intravenous cisplatin at a dose of 35 mg/m^2^ every 7 days, starting on day 1 of radiotherapy, was administered to all participants.

### MRI Imaging

Participants underwent MRI before treatment and at 6 and 12 weeks after completion of chemoradiotherapy. Imaging was performed on a 1.5-Tesla MRI system (MAGNETOM Aera, Siemens Healthcare, Erlangen, Germany) using a 20-channel phased-array surface neck coil and included T2-weighted, diffusion-weighted imaging (DWI) and post gad T1w sequences. The MRI acquisition protocol is summarised in **Supplemental Table 1**.

### MRI Processing and Analysis

**Figure 1:**
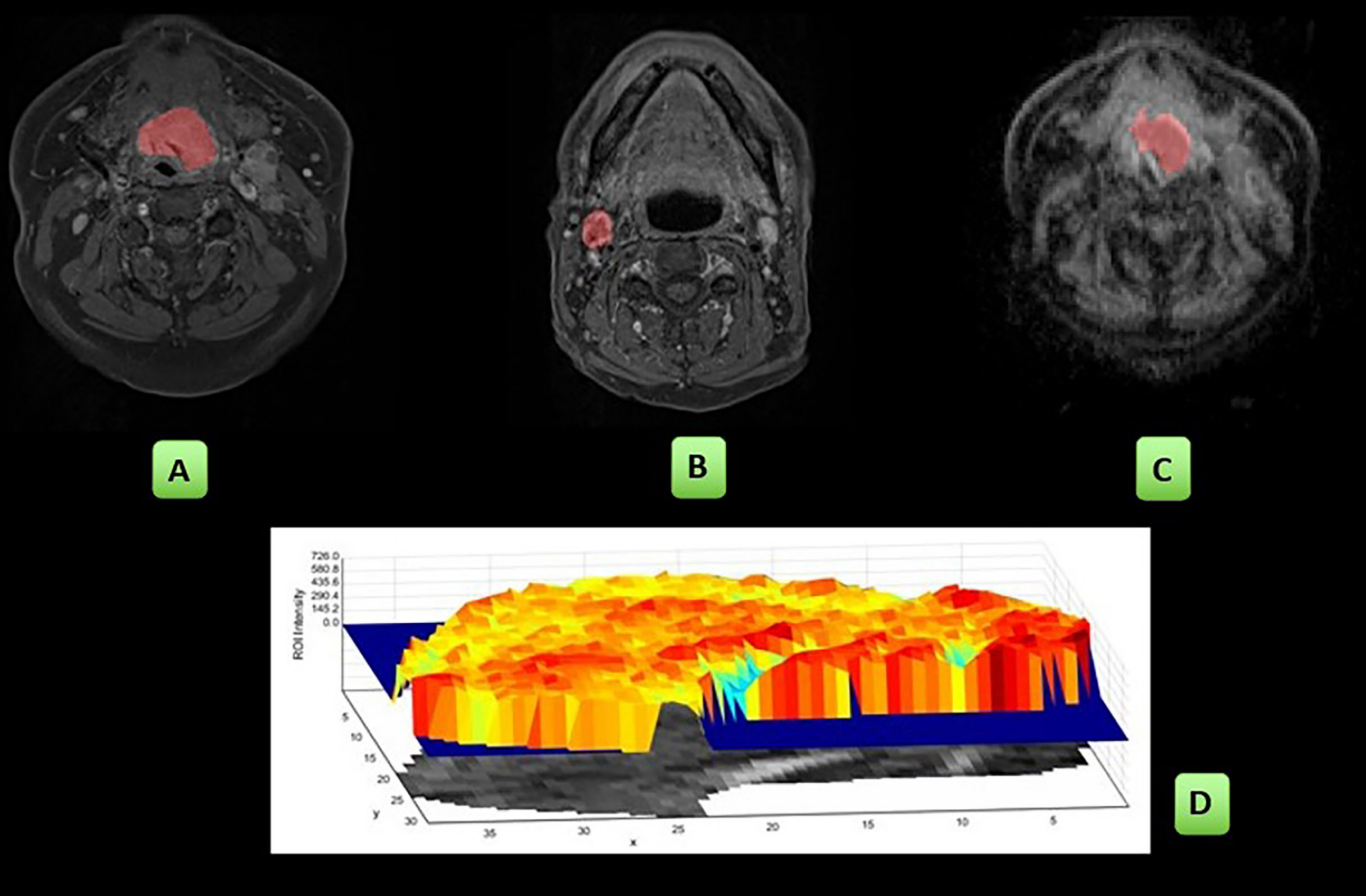
Workflow of the study: ROIs were placed on the post gad T1w sequences: primary tumour **(A)**, single largest pathological lymph node **(B)** and on the ADC map: primary tumour **(C)**. The radiomics first- and second-order parameters were derived and are pictorially depicted here by a sample of the shaded surface intensity histogram **(D)**. These analyses were then repeated at the 6- and 12-week MRI studies.

Two sets of freehand regions of interest (ROI) were delineated by a radiologist (AG, 7 years of experience) on both DWI (b = 800) and post gad T1w images with reference to the other sequences. ROIs were placed at the site of the measurable primary tumour and/or largest pathological lymph node on the pretreatment, 6-week and 12-week post-treatment MRIs. ROIs were placed on multiple sections to encompass the whole lesion depicted as high signal on the DWI b-800 or high signal on post gad T1-w sequences (**Figure 1**). Internal areas of necrosis (non-enhancement and high diffusion signal, respectively) were included, but areas of peri-tumoural inflammation (adjacent avid gadolinium enhancement and high signal on T2w images) were excluded. When a focus of increased signal relative to background was not evident on the post-CRT imaging, this was defined as non-measurable, and a representative ROI (6-mm diameter/28 mm^2^) was placed at the site of the pretreatment ROI. For DWI, the ROI was transferred onto the ADC maps calculated from the b = 100 and b = 800 values to derive ADC parameters. In patients where it was challenging to draw ROIs on the posttreatment scans, the T2W and T1 post-gadolinium images were also correlated with ensuring the accuracy of delineation.

Primary tumour and nodal histogram and grey-level co-occurrence matrix (GLCM) parameters were extracted from the ROIs using an in-house radiomics software platform, validated through the International Biomarker Standardization Initiative (14). Analysis was restricted to these parameters to reflect the limitations of the 2D acquisition and in-plane voxel resolution. For example, GLCM analysis was only applied to post gad T1w images since the larger voxel size (0.9 × 0.9 × 5 mm) of the diffusion acquisition resulted in insufficient voxels for analysis.

The mean and median gadolinium-enhanced T1w signal and ADC value were also recorded as well as the lesion long axis length and volume on the post gad T1w images.

### Clinical Data and Treatment Outcome

Patient demographics, TN status, human papillomavirus (HPV) status, tumour site and subsite tumour stage were recorded. Follow-up included a standard-of-care 12-week 18F-FDG PET/CT study which was used to guide management and clinical assessment at 1 year and 2 years following completion of chemo-radiotherapy. The response of primary tumour and largest lymph node at 2 years was determined by cytological or histological confirmation (biopsy or resection) or by serial progression on imaging follow-up.

### Statistical Analyses

SPSS statistical software Version 21.0 was used for analysis (IBM SPSS Statistics for Windows, Version 21.0. Armonk, NY: IBM Corp. Released 2012).

Pearson’s correlation was performed as a method of parameter reduction amongst the initial 21 GLCM parameters, excluding those with a high correlation coefficient (>0.75). This left 4 GLCM parameters for further analysis (**Supplemental Table 2**).

Descriptive data were collated for pretreatment, 6-week and 12-week post-CRT histogram and the selected GLCM parameters. This was performed separately for primary tumour and lymph node values and according to whether there was treatment response at 2 years.

As variables were not normally distributed, the Wilcoxon signed-rank test was performed to assess the interval changes in heterogeneity parameters. A p-value of p < 0.001 was considered statistically significant to account for multiple testing.

## Results

### Participants

**Figure 2:**
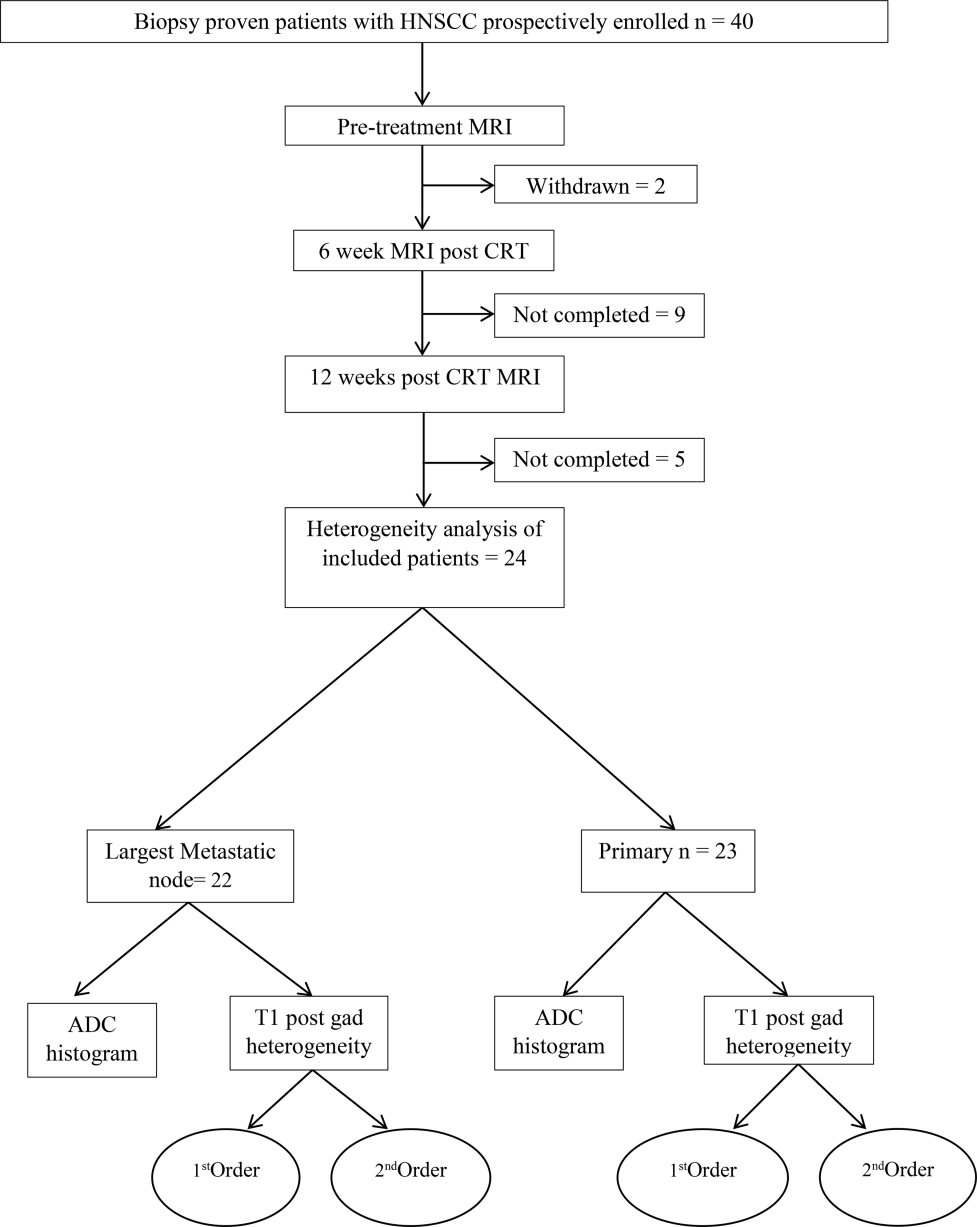
Patient flowchart.

The participant consort flow diagram is demonstrated in **Figure 2**. Of the 24 participants (20 men, 4 women, mean age 62.9 +_8.79 years), there were 5 (20%) patients with stage III disease and 19 (79%) patients with stage IV disease. Participant characteristics including primary site, HPV status and TN staging are summarised in **Table 1**. There were 23 primary tumours and 22 lymph nodes analysed.

**Table 1:** Participant characteristics of the HNSCC cohort.

**Gender**	Male	20
	Female	4
**Age (years)**	Mean	62.95
	Median	65
	Range	47–77
**Tumour location**	Oropharynx (all HPV positive)	17
	Hypopharynx	3
	Laryngopharynx	4
**T stage**	T1	5
	T2	7
	T3	6
	T4	6
**N stage**	N0	2
	N1	5
	N2a	1
	N2b	12
	N2c	4
Human papillomavirus status	Positive	21
	Negative	3

At 6 weeks, there were 19/23 non-measurable primary tumours and 10/22 non-measurable nodes whilst at 12 weeks there were 23/23 non-measurable primary tumours and 18/22 non-measurable nodes. All primary tumours and 18/22 lymph nodes responded to CRT at 2 years. The 4 cases of lymph node recurrence corresponded to the same largest lymph node site as that undergoing analysis. Patients with non-responding lymph nodes underwent salvage neck dissection following initial cytological assessment.

### Interval Changes Following CRT in the Primary Tumour

All primary tumours responded to CRT at 2 years following treatment.

The primary tumour showed a significant mean decrease of 71% (6.3+_0.8 cm) in diameter and 98% (1.45+_0.5 cm^3^) in volume at 6 weeks and a further reduction between 6 and 12 weeks (p <.0001).

There was a significant increase in mean values of ADCmean and ADCmedian (729 to 1,019 × 10^-6^ mm^2^/s; 40% and 692 to 1,062 × 10^-6^ mm^2^/s; 53%) by 6 weeks post CRT, but there was no further significant increase between 6 and 12 weeks. Kurtosis (-42%), coefficient of variation (CoV) (-46%) and entropy (-44%) significantly decreased, and energy significantly (+238%) increased by 6 weeks of treatment (p <.0001), again without further significant alterations between 6 and 12 weeks post CRT (**Table 2**).

**Table 2:** Pretreatment, 6-week post-CRT and 12-week post-CRT primary tumour ADC and post-gadolinium T1w histogram parameters and their interval changes with treatment (all with treatment success n = 23).

Primary tumour (n = 23)	Pretreatment	6 weeks post therapy	12 weeks post therapy	% change pretreatment to 6 weeks	p-value	% change pretreatment to 12 weeks	p-value	% change 6 to 12 weeks	p-value
**Parameter**	**Median (IQR)**	**Median (IQR)**	**Median (IQR)**						
Diameter (cm)	2.36	0.51	0.66	-71	**<0.001**	-71	**<0.001**	-2	0.07
	(1.756–2.71)	(1.12–0.61)	(0.59–1.74)						
Volume (cm^3^)	6.91	0.07	0.15	-97	**<0.001**	-98	**<0.001**	-6	0.07
	(316.64–533.74)	(0.75–0.11)	(0.11–2.76)						
**ADC (× 10^-6^ mm^2^/s)**									
Mean	729.10	1019.68	1165.74	+40	**<0.001**	+60	**<0.001**	+14	0.12
			(1006.66–1197.93)						
Median	692.75	1062.5	1169.25	+53	**<0.001**	+69	**<0.001**	+10	0.11
Skewness	0.30	–0.024	0.06	-108	0.02	-78	0.05	-370	0.18
Kurtosis	3.52	2.05	1.99	-42	**<0.001**	-43	**<0.001**	-3	0.21
CoV	0.23	0.12	0.10	-46	**<0.001**	-54	**<0.001**	-16	0.63
Entropy	4.26	2.39	2.50	-44	**<0.001**	-41	**<0.001**	+5	0.30
Energy	0.06	0.21	0.18	+238	**<0.001**	+192	**<0.001**	-14	0.19
**T1w post-gadolinium signal intensity**									
Mean	430.27(316.648–533.74)	416.06	437.457	-3	0.25	+2	0.73	+5	0.2
		(320.99–496.55)	(365.065–503.834)						
Median	439.5	408.25	433.5	-7	0.31	-1	0.77	+6	0.14
	(303.25–532.50)	(321.00–489.25)	(370.62–503.87)						
Skewness	0.05	0.09	0.11	+66	0.78	+93	0.95	+17	0.76
	(–0.43–0.51)	(–0.39–0.32)	(–0.09–0.39)						
Kurtosis	3.24	2.78	2.75	-14	0.19	-15	0.10	-1	0.73
	(2.67–3.55)	(2.43–3.02)	(2.46–3.29)						
CoV	0.19	0.11	0.12	-41	0.05	-36	0.05	+8	0.64
	(0.11–0.22)	(0.10–0.18)	(0.09–0.16)						
Entropy	4.14	4.41	4.37	+6	0.11	+5	0.01	-1	0.28
	(3.93–4.30)	(3.99–4.50)	(4.23–4.52)						
Energy	0.068	0.05	0.05	-16	0.07	-16	0.01	+0	0.18
	(0.06–0.08)	(0.05–0.07)	(0.05–0.06)						
GLCM contrast	144.30	297.42	291.61	+106	**<0.001**	+102	**<0.001**	-2	0.31
	(91.13–177.02)	(230.82–441.22)	(250.77–338.30)						
GLCM autocorrelation	1141.86	1051.333	971.88	-8	0.82	-15	0.02	-8	0.46
	(1093.48–1170.03)	(879.078–1403.38)	(873.51–1091.60)						
GLCM entropy	6.79	4.35	4.35	-36	**<0.001**	-36	**<0.001**	+0	0.28
	(6.46–7.08)	(3.66–4.71)	(4.19–4.49)						
GLCM cluster shade	203.63	1010.87	1402.37	+396	0.99	+589	0.93	+39	0.68
	(–2503.10–3129.10)	(–4628.71–5465.97)	(11.05–4029.17)						

p<0.001 considered statistically significant.

None of the histogram parameters showed any significant changes on post gad T1w images at 6 or 12 weeks. GLCM contrast significantly increased (+106%) and GLCM entropy (-36%) significantly decreased by 6 weeks, with no further significant change between 6 and 12 weeks post CRT (**Table 2**).

### Interval Changes Following CRT in the Lymph Nodes

There were 18/22 lymph nodes that responded to CRT at 2 years following treatment.

The responding nodes showed a significant decrease in the mean diameter of 68% (1.4+_0.3) and volume of 95% (8+_1.4) at 6 weeks and a further reduction between 6 and 12 weeks (p <.0001).

There was no significant increase in mean values of ADCmean and ADCmedian by 6 or 12 weeks post CRT.

ADC kurtosis showed a significant mean decrease (-35%) at 6 weeks. By 12 weeks, energy (+170%) and CoV (+280%) showed a significant mean increase compared to the pretreatment values whilst there was a statistically significant mean reduction in entropy (-39%).

None of the post gad T1w histogram parameters showed significant change with treatment. However, mean GLCM contrast significantly increased and mean GLCM entropy significantly decreased (+61% and -20%) by 6 weeks post CRT with no further significant alteration between 6 and 12 weeks (**Table 3**).

**Table 3:** Pretreatment, 6-week post-CRT and 12-week post-CRT ADC and post-gadolinium T1w texture parameters and their interval changes in metastatic lymph nodes of participants with treatment response (n = 18).

Largest node (n = 18)	Pretreatment	6 weeks post therapy	12 weeks post therapy	% change pretreatment to 6 weeks	p-value	% change pretreatment to 12 weeks	p-value	% change 6 to 12 weeks	p-value
**Parameter**	**Median (IQR)**	**Median (IQR)**	**Median (IQR)**						
Diameter (cm)	1.92	0.60	0.58	-69	**<0.001**	-70	**<0.001**	-4	1
	(1.45–2.79)	(0.59–1.03)	(0.59–0.68)						
Volume (cm^3^)	3.74	0.19	0.11	-95	**<0.001**	-96	**<0.001**	-39	1
	(1.61–11.46)	(0.11–0.58)	(0.10–0.17)						
**ADC (mm^2^/s)**									
Mean	745.42	824.68	980.27	+11	0.30	+32	0.01	+19	0.12
	(634.19–957.6)	(686.46–971.37)	(868.44–1130.14)						
Median	821.08	856.5	972.5	+4	0.17	+18	0.01	+14	0.11
	(0.19–0.34)	(1.86–2.58)	(821.12–1126.50)						
Skewness	0.30	0.06	–0.02	-78	0.01	-108	0.10	-138	0.18
	(–0.10–0.72)	(–0.19–0.31)	(–0.28–0.21)						
Kurtosis	3.60	2.35	2.19	-35	**<0.001**	-39	**<0.001**	-7	0.21
	(2.96–5.22)	(1.93–2.87)	(1.86–2.58)						
CoV	0.23	0.12	0.10	+60	0.01	+280	**<0.001**	-16	0.63
	(0.19–0.29)	(0.09–0.19)	(0.09–0.13)						
Entropy	4.09	3.26	2.50	-20	0.01	-39	**<0.001**	-23	0.30
	(3.74–4.37)	(2.50–4.12)	(2.17–3.12)						
Energy	0.06	0.18	0.21	+170	0.01	+170	**<0.001**	+14	0.187
	(0.05–0.10)	(0.16–0.22)	(0.18–0.24)						
**T1w post-gadolinium signal intensity**									
Mean	435.8	441.74	502.53	+1	0.72	+15	0.07	+14	0.19
	(363.51–510.12)	(341.51–578.21)	(308.77–537.53)						
Median	435.9	443.68	506.39	+2	0.81	+16	0.07	+14	0.19
	(347–52)	(334.50–584.00)	(307.25–537.75)						
Skewness	–0.14	–0.007	0.08	-95	0.42	+39	0.81	+1342	0.88
	(–0.74–0.38)	(–0.56–0.51)	(–0.16–0.23)						
Kurtosis	3.4	3.30	3.23	-3	0.72	-4	0.58	-2	0.83
	(2.37–4.12)	(2.56–3.5)	(2.588–3.7)						
CoV	0.71	0.78	0.93	+10	0.00	+31	0	+19	0.47
	(0.67–0.70)	(0.69–0.84)	(0.74–0.94)						
Entropy	4.2	4.41	4.31	+5	0.03	+3	0.07	-2	0.19
	(4.03–4.39)	(4.205–4.62)	(3.88–4.45)						
Energy	0.06	0.05	0.06	-44	0.04	-14	0.05	+7	0.20
	(0.05–0.07)	(0.04–0.06)	(0.05–0.08)						
GLCM contrast	145.5	234.89	264.33	+61	**<0.001**	+82	**<0.001**	+13	0.46
		(157.75–319.80)							
GLCM autocorrelation	1114.34	1146.76	1168.3	-3	0.34	-5	0.06	+2	0.73
	(1054.0–1161.4)	(893.15–1398.21)	(893.15–1398.21)						
GLCM entropy	6.96	5.55	4.42	-20	**< 0.001**	-36	**<0.001**	-20	0.002
	(6.60–7.18)	(4.48–5.96)	(4.19–4.57)						
GLCM cluster shade	404.66	385.24	584.18	-195	0.44	+244	0.62	+52	0.58
	(–4224.62–2081.77)	(–2066.94–2792.61)	(–2234.55–2116.94)						

p<0.001 considered statistically significant.

The non-responding metastatic lymph nodes did not demonstrate a significant change in any ADC or post gadolinium T1w histogram or GLCM parameters between pretreatment and post-CRT studies. They also did not show a significant decrease in diameter or volume (**Table 4**).

**Table 4:** Pretreatment, 6-week post-CRT and 12-week post-CRT ADC and post-gadolinium T1w histogram parameters and their interval changes in metastatic lymph nodes of participants with treatment failure (n = 4).

Node non-responders (n = 4)	Pretreatment	6 weeks post therapy	12 weeks post therapy	% change pretreatment to 6 weeks	p-value	% change pretreatment to 12 weeks	p-value	% change 6 to 12 weeks	p-value
**Parameter**	**Median (IQR)**	**Median (IQR)**	**Median (IQR)**						
Effective diameter (cm)	2.2	1.1	0.6	-50	0.14	-71	0.14	-45	0.14
	(1.23–3.5)	(0.69–1.55)	(0.50–1.35)						
Volume (cm^3^)	8.6	0.9	0.1	-90	0.14	-98	0.14	-89	0.12
	(1.35–19.59)	(0.19–2.11)	(0.07–1.65)						
**ADC (mm^2^/s)**									
Mean	687	932	949.2	36	0.14	57	0.14	2	0.62
	(580.90–876.15)	(647.52–1119.22)	(848.36–1208.84)						
Median	698.3	918.3	969.3	32	0.14	53	0.14	6	0.87
	(576.00–884.25)	(640.25–1134.50)	(835.38–1203.63)						
Skewness	0.6	–0.1	0.3	-117	0.46	-400	0.46	-400	0.62
	(–0.22–2.23)	(–0.69–0.42)	(–0.46–0.66)						
Kurtosis	5.9 (2.21–12.76)	2.8 (2.00–3.70)	2.3 (2.08–2.81)	-53	0.71	-18	0.27	-18	0.62
CoV	0.3 (0.17–0.55)	0.2 (0.17–0.26)	0.218 (0.14–0.24)	-33	0.46	-8	0.27	9	0.62
Entropy	4.1 (3.52–4.49)	3.7 (2.78–4.29)	2.0 (1.52–4.08)	-10	0.14	-52	0.14	-46	0.25
Energy	0.06	0.08	0.3	60	0.14	200	0.14	275	0.25
	(0.05–0.12)	(0.06–0.18)	(0.08–0.37)						
**T1w post-gadolinium signal intensity**									
Mean	473.98	436.02	442.05	-2	0.71	-1	1	1	0.87
	(276.67–548.19)	(244.55–595.17)	(308.77–537.53)						
Median	499.5	440	439	-4	0.71	-4	1	0	0.87
	(277.25–557.50)	(243.25–598.50)	(307.25–537.75)						
Skewness	0.09	–0.1	0.08	-12	0.71	-186	0.71	-198	0.61
	(–0.50–0.23)	(–0.27–0.22)	(–0.16–0.23)						
Kurtosis	2.83	2.82	3.34	-1	0.71	6	0.46	7	0.14
	(2.44–3.85)	(2.74–3.46)	(2.58–3.72)						
CoV	0.81	0.78	0.83	-4	0.71	2	0.46	6	0.14
	(0.67–47.90)	(0.69–0.84)	(0.744–0.94)						
Entropy	4.26	4.43	4.28	4	0.47	-1	0.71	-5	0.87
	(3.99–4.47)	(4.23–4.56)	(3.88–4.45)						
Energy	0.06	0.05	0.06	-13	0.47	3	1	18	0.72
	(0.05–0.07)	(0.04–0.06)	(0.05–0.080						
GLCM contrast	0.97	4.58	300.09	+372	0.07	+30837	0.07	+6452	0.07
	(0.84–1.18)	(4.34–5.22)	(168.86–412.19)						
GLCM autocorrelation	6.64	973.99	4.37	+14,555	0.07	-34	0.14	-100	0.07
	(5.96–6.98)	(879.09–1152.96)	(4.18–6.05)						
GLCM entropy	473.98	436.02	442.05	+841	0.07	-99	0.07	-100	0.07
	(276.67–548.19)	(244.55–595.17)	(308.77–537.53)						
GLCM cluster shade	499.5	440	439	-126	0.07	-9	1	-456	0.07
	(277.25–557.50)	(243.25–598.50)	(307.25–537.75)						

## Discussion

### Summary of Findings

Interval changes in signal heterogeneity have been evaluated to provide insight into the effect of CRT on the primary tumour and lymph nodes in HNSCC, and to identify whether heterogeneity changes might provide an earlier indication of treatment response. Changes in selected histogram parameters were observed by 6 weeks on ADC maps in responding primary tumours (kurtosis, CoV, entropy, energy) and responding lymph nodes (kurtosis). Whilst there were no post CRT changes in histogram parameters on post gad T1w images, there were significant decreases in GLCM entropy for both successfully treated primary tumours and lymph nodes. The non-responding lymph nodes did not demonstrate any of these interval changes in heterogeneity. Interval reduction in size was also demonstrated by 6 weeks in responding lymph nodes but not in non-responding lymph nodes.

### ADC

Previous studies have evaluated the role of diffusion-weighted MRI in predicting HNSCC treatment response post CRT, and most have found that a greater rise in ADCmean from the pretreatment to the intra-treatment (15–19) or posttreatment MRI (20, 21) predicts treatment outcomes. Similarly, our study demonstrated that primary tumour ADCmean increased by 6 weeks post CRT. However, interval changes in ADCmean did not differ between metastatic lymph nodes that did and did not respond to treatment. This may be due to our inclusion of necrotic areas in the ROIs, which could have led to an overestimation of baseline ADCmean.

King et al. found that ADC histogram analysis could predict intra-treatment response at the primary site as early as 2 weeks after the start of treatment, with primary tumours showing higher histogram skewness (20). de Parrot et al. demonstrated that the ADC histogram kurtosis ratio (22) was useful in differentiating histologic grades of HNSCC at b = 2,000 s/mm^2^. A general shift in skewness to the left means that the tail of the ADC curves got heavier toward the negative and signifies a greater asymmetry of the data posttreatment. Similar findings were reported by Forouton et al. (23), who demonstrated negative skewness and greater asymmetry following therapy in the treatment group when compared to the control group. Whilst our data showed a non-significant increase in skewness post CRT, the histogram parameters of kurtosis, coefficient of variation and energy were found to demonstrate a significant increase. This was demonstrated by 6-week post CRT for primary tumour and 12 weeks post CRT for lymph nodes. The underlying histopathological basis for this is yet to be studied; however, it appears there is a trend toward greater homogeneity of the tumour with treatment.

### Post-Gadolinium T1w MRI

There are limited data on the interval changes in macroscopic T1w gadolinium enhancement following CRT for HNSCC. A qualitative grading (24) showed that changes in the pattern of necrosis on T1w gadolinium enhancement imaging could predict the outcome. The GLCM is a tabulation of how often different combinations of grey levels occur between neighbouring pixels in an image (21–23). Reinert et al. (25) found that GLCM entropy significantly decreased, whereas GLCM uniformity significantly increased (p   < .001) after therapy in a study assessing response of Hodgkin lymphoma to chemotherapy. Whilst none of the histogram parameters changed significantly post CRT in our cohort, GLCM contrast increased whilst GLCM entropy decreased with treatment in both the primary tumour and lymph node by 6 weeks post CRT. It is of interest that changes were observed in similar parameters in both metastatic lymph nodes (largely non-enhancing necrosis in 16/18 pretreatment) and primary tumours (homogenously enhancing in 22/23 pretreatment) considering the marked difference in pretreatment enhancement patterns.

### Differences Between the Primary Tumour and Nodes

The interval changes in ADC-based histogram parameters differed between the primary tumours and metastatic lymph nodes, with earlier changes being observed with primary tumours. Although there are no previous comparable data available for ADC-based histogram parameters, a potential disparity between the timing and nature of posttreatment ADC-related biomarkers between the primary tumour and nodes has been established in previous studies. In particular, these have demonstrated that the ADCmean acquired from nodal sites is able to predict treatment outcomes, whereas ADCs acquired from primary sites do not (26), whilst predictive threshold ADC measurements differ between primary tumour and nodal sites (27).

### Comparison With Primary Tumour and Nodal Size and Volume Change

The assessment of morphological features such as changes in tumour dimensions has provided prognostic stratification (20, 24, 28, 29) in the posttreatment setting, but they have variable diagnostic accuracy in determining treatment response (30, 31). A significant reduction in lymph node dimensions at 6 weeks post CRT was only shown in those with successful treatment. However, it is of interest that the difference in magnitude between responding and non-responding lymph node dimensions was less than that demonstrated with some heterogeneity parameters. The decrease in diameter and volume of the responding lymph nodes at 6 weeks post CRT was 69% and 95% as compared to 50% and 90% with the non-responding lymph nodes. Thus, the addition of histogram parameters could perhaps aid in increasing the discriminatory power of size criteria in assessing posttreatment changes, even as early as 6 weeks posttreatment.

### Limitations

It is acknowledged that there are some limitations to this study.

Firstly, it should be noted that the cohort was largely represented by HPV-positive oropharyngeal cancer. HPV-OPC is a clinically and histologically distinct form of HNSCC which has a better outcome irrespective of treatment choice. Since it exhibits particular histopathological features such as indistinct cell borders and comedo-necrosis, there should be caution in extrapolation of the results to a wider HNSCC population.

Secondly, there was only a small sample of non-responders to CRT with only four metastatic lymph nodes and no primary tumours which did not respond to treatment. The small sample of non-responding lymph nodes precluded a direct statistical comparison of parameters between responding and non-responding lymph nodes. It also raises the possibility that interval changes in histogram and GLCM parameters in the non-responding lymph nodes were not detected due to a type 2 error. Future studies should be conducted with a larger HPV negative cohort and a greater proportion of non-responders such that these texture parameters can be evaluated for their ability to predict treatment response.

Finally, it could be argued that the outcomes were influenced by our method of ROI analysis which was focused on whole volumes of the primary tumour or lymph node. Both volume and area-based ROI data are variably presented in the literature. However, there are limited data on how this impacts on histogram or GLCM analysis. Generous sampling of the whole volume may better reflect tumour heterogeneity. However, smaller ROIs benefit from the ability to exclude macroscopic necrosis. It is possible that sampling certain parameters such as ADCmean may have revealed significant changes if confined to enhancing “viable tumour”, particularly with respect to the largely necrotic lymph nodes.

## Conclusions

This exploratory study indicates that significant post-CRT interval changes in histogram and GLCM parameters are demonstrated on ADC maps and T1w post gad images as early as 6 weeks following successful treatment for stage III and IV HNSCC. The selection of appropriate ADC map histogram parameters may differ, depending on whether biomarkers for primary tumour or lymph node response are being evaluated. A small sample of non-responding metastatic lymph nodes did not demonstrate such interval evolution of these heterogeneity parameters; however, standard size criteria were also able to predict treatment failure in these patients.

## Data Availability Statement

The data that support the findings of this study are available from the corresponding author upon reasonable request.

## Ethics Statement

Institutional approval from the Research Ethics Committee (REC reference 13/LO/1876) and informed consent was obtained from all participants. The study conforms to recognized standards of the Declaration of Helsinki.

## Author Contributions

Conceptualization, SC, VG; Methodology, SC, VG, GC; Investigation, SC, AG, MA; Data Curation, AG, SC; Writing - Original Draft, AG, SC; Writing - Review & Editing, VG, GC; Supervision, SC, VG; Project Administration, SC: Funding Acquisition, SC.

## Funding

This research was funded in whole, or in part, by the Wellcome Trust [203148/Z/16/Z]. For the purpose of open access, the author has applied a CC BY public copyright licence to any Author Accepted Manuscript version arising from this submission. Authors acknowledge funding support from Wellcome/Engineering and Physical Sciences Research Council Centre for Medical Engineering at King’s College London (WT 203148/Z/16/Z); National Institute for Health Research Biomedical Research Centre at Guy’s & St Thomas’ Hospitals and King’s College London; Cancer Research UK National Cancer Imaging Translational Accelerator (A27066); the UK Research & Innovation London Medical Imaging and Artificial Intelligence Centre. Authors also acknowledge funding suport from Guy’s and St Thomas’ Hospital Charity (ref EFT130501) and the Royal College of Radiologists: Kodak Radiology Fund Research Bursary.

## Conflict of Interest

The authors declare that the research was conducted in the absence of any commercial or financial relationships that could be construed as a potential conflict of interest.

## Publisher’s Note

All claims expressed in this article are solely those of the authors and do not necessarily represent those of their affiliated organizations, or those of the publisher, the editors and the reviewers. Any product that may be evaluated in this article, or claim that may be made by its manufacturer, is not guaranteed or endorsed by the publisher.
