# Supplementary material for: Radiomic Analysis of Tumour Heterogeneity Using MRI in Head and Neck Cancer Following Chemoradiotherapy: A Feasibility Study
Source: Front Oncol. 2022 Feb 15;12:784693. doi: 10.3389/fonc.2022.784693 (PMC8886142; doi:10.3389/fonc.2022.784693)
Supplement: Supplementary file 1 [file DataSheet_1.docx]

MRI protocol:

Participants underwent full standard institutional head and neck protocol on a 1.5 Tesla MRI system (Magnetom Aera, Siemens Healthcare, Erlangen, Germany) using a surface phased array 20 channel neck coil (table 1) In addition, a research echo planar diffusion-weighted sequence was acquired including matched images in the axial plane with multiple b-values (0, 50, 100, 800 and 1500 s/mm^2^) with ADC maps calculated from the b=100 and b=800 values (Supplementary table 1).

Supplementary Table 1. Summary of MRI acquisition parameters

|  | Slice thickness/ gap | TR/TE | Field of view | Number of averages | Pixel Bandwidth | Flip angle | Acquisition matrix |
| --- | --- | --- | --- | --- | --- | --- | --- |
| T1w  Axial | 4/0 | 549/11 | 220x220 | 1 | 200 | 160 | 384/269 |
| T2w Axial | 4/0 | 5830/102 | 220x220 | 1 | 190 | 150 | 384/346 |
| DWI^+^ Axial | 4/0.5 | 5900/60 | 240x240 | 2 | 1375 | 90 | 130 x130 |
| T1w fat saturated  post- gad* Axial | 4/0 | 566/11 | 220x220 | 1 | 330 | 145 | 320x224 |
| STIR  Coronal | 3/0.3 | 3000/35  TI 140 | 260X260 | 1 | 220 | 160 | 320X224 |
| T1w fat saturated post-gad* Coronal | 3/0.3 | 708/10 | 280X280 | 1 | 340 | 145 | 320X320 |
| *0.1 mmol gadolinium/kg body weight and at an injection rate of 1.5 ml/s | | | | | | | |

Supplementary figure 1: Heat map demonstrating the Pearson’s correlation matrix of metastatic node parameters at baseline scan

Supplementary figure 2: A heat map demonstrating the Pearson’s correlation matrix of primary tumour parameters at pretreatment scan
